# Development of Imperatorin Nanostructured Lipid Carriers with Grape Seed Oil for Boosting Oral Absorption and Antioxidant Capacity

**DOI:** 10.3390/molecules31152605

**Published:** 2026-07-26

**Authors:** Haonan Qiu, Li Zhang, Yu Zhang, Chi Zhang, Chunfei Wang, Lutan Zhou, Xiu Wang, Lihua Li, Xuefeng Hou

**Affiliations:** 1 Drug Research and Development Center, School of Pharmacy, Wannan Medical University, Wuhu 241002, China; 20249157@stu.wnmc.edu.cn (H.Q.); zhangli@stu.wnmc.edu.cn (L.Z.); 20249171@stu.wnmc.edu.cn (Y.Z.); 22107070024@stu.wnmc.edu.cn (C.Z.); 20220028@wnmc.edu.cn (C.W.); 20210007@wnmc.edu.cn (L.Z.); 2 Anhui Engineering Technology Research Center of Biochemical Pharmaceutical, Bengbu Medical University, Bengbu 233030, China; 3 Anhui Provincial Engineering Laboratory for Screening and Re-Evaluation of Active Compounds of Herbal Medicines in Southern Anhui, Wannan Medical University, Wuhu 241002, China; 4 Anhui Provincial Engineering Research Center for Polysaccharide Drugs, Wannan Medical University, Wuhu 241002, China; 5 Anhui Innovative Center for Drug Basic Research of Metabolic Diseases, Wannan Medical University, Wuhu 241002, China

**Keywords:** imperatorin, nanostructured lipid carriers, grape seed oil, oral bioavailability, uptake mechanism, intestinal absorption

## Abstract

Imperatorin (IPT) is a naturally occurring coumarin with recognized antioxidant and anti-aging properties; unfortunately, its poor water solubility and low oral bioavailability severely limit its practical use. To get around these issues, we formulated IPT-loaded NLCs using grape seed oil and glyceryl monostearate—both food-grade excipients—with the goal of enhancing oral absorption. Optimized IPT@NLCs were prepared by high-pressure homogenization, featuring uniform spherical morphology, an average particle size of 186.63 ± 1.65 nm, a PDI of 0.188 ± 0.008, an encapsulation efficiency of 99.54 ± 0.10%, and a drug loading capacity of 9.08 ± 0.23%. IPT@NLCs remained stable in SGF, while their cumulative in vitro release over 48 h reached 90.56 ± 3.12% in SIF. We established a Caco-2/HT29-MTX-E12 co-culture monolayer to examine mucus penetration, cellular uptake, and transcellular transport routes. In parallel, oxidative stress experiments using 3T3-L1 cells, along with in vivo pharmacokinetic and gastrointestinal safety evaluations, were conducted to provide complementary evidence. Our results indicate that NLC encapsulation significantly improves both the dissolution and intestinal uptake of IPT, primarily by shifting the absorption mechanism from passive diffusion to energy-dependent active transport. In addition, IPT@NLCs effectively reduce intracellular oxidative damage through modulation of endogenous antioxidant enzyme activities. Animal studies further reveal an approximately 9-fold increase in relative oral bioavailability, with no notable irritation to gastrointestinal tissues. Overall, GSO-based NLCs offer safe and efficient oral delivery, enhancing IPT bioavailability and antioxidant activity, providing a strategy for developing natural-product-based formulations.

## 1. Introduction

Imperatorin (IPT) is the main coumarin ingredient isolated from *Angelicae Dahuricae Radix*, a Chinese herbal material that is used as both a medicine and a food [1,2,3,4]. Recent pharmacological studies have confirmed that IPT exhibits a broad range of activities, including analgesic, anti-inflammatory, antimicrobial, antioxidant, and immunomodulatory effects, underscoring its potential in therapeutic and health-related applications [5,6]. Moreover, its low toxicity and good safety record make it suitable for developing dietary supplements, wellness products, and novel oral formulations [7]. Despite these pharmacological merits, IPT’s practical use is hampered by its poor physicochemical properties. Because IPT is highly lipophilic, its water solubility is extremely low, which inevitably leads to poor intestinal absorption and low oral bioavailability [8,9]. From a manufacturing standpoint, this drawback complicates the production of oral dosage forms and functional foods, compromises in vivo efficacy, and ultimately hinders large-scale production and market expansion.

Oral administration remains the most widely used route for both drugs and nutraceuticals, thanks to its non-invasive nature, convenience, low cost, and high patient/consumer acceptance [10,11]. Nevertheless, the complex physiological microenvironment of the gastrointestinal (GI) tract imposes multiple inherent biological barriers, which severely hinder the oral absorption and in vivo efficacy of lipophilic active molecules represented by IPT, becoming the core factor leading to its low oral bioavailability [12,13]. Specifically, the harsh acidic environment of the stomach can trigger structural degradation and inactivation of active ingredients, while abundant GI digestive enzymes further metabolize and decompose drugs, causing massive loss of effective components prior to systemic absorption [14,15]. Beyond chemical and metabolic barriers, the intestinal mucus layer acts as a crucial physical barrier restricting oral drug delivery. As a dense, negatively charged porous layer covering the intestinal epithelial surface, intestinal mucus readily traps hydrophobic compounds via hydrophobic interactions and molecular entanglement, preventing lipophilic IPT from penetrating the mucosal layer and contacting intestinal epithelial cells [16]. Furthermore, the compact intestinal epithelial tight junctions further limit transmembrane penetration of poorly permeable lipophilic components, while pre-systemic GI clearance collectively reduces the effective drug concentration reaching systemic circulation and target tissues [17]. To compensate for low absorption efficiency, excessive drug dosage is commonly adopted in conventional oral formulations, which fails to fundamentally improve drug utilization and may induce potential in vivo toxicological risks and safety concerns [18].

Nanostructured lipid carriers (NLCs), as an optimized novel lipid nano-delivery system developed on the basis of traditional solid lipid nanoparticles, can effectively break through the aforementioned multi-layered oral biological barriers and perfectly compensate for the inherent defects of IPT [19]. Different from the single solid lipid matrix of SLNs, NLCs feature a dual composite lipid framework composed of solid lipids and liquid lipids, forming an imperfect lipid crystal network internally [20]. This unique structural characteristic endows NLCs with superior drug loading capacity and excellent physicochemical stability, effectively avoiding drug leakage and crystal precipitation during GI transit and storage [21,22]. A key advantage of NLCs is their ability to tackle different oral absorption barriers in a stepwise, barrier-appropriate fashion. To begin with, the outer lipid shell protects the encapsulated IPT from both gastric acid and digestive enzymes, thereby preserving the compound’s structural integrity and bioactivity as it passes through the upper gastrointestinal tract [23]. Thanks to their submicron particle size and moderately hydrophilic surfaces, NLCs are not readily trapped or retained by the viscous intestinal mucus; instead, they diffuse quickly through the mucus layer and accumulate efficiently at the epithelial surface [24]. Once past the mucus, the lipid-compatible nature of NLCs promotes close interaction with intestinal epithelial membranes, which in turn supports efficient transcellular transport and cellular uptake, effectively bypassing the permeability barrier [25]. Meanwhile, the greatly improved water dispersibility of NLCs resolves the poor aqueous solubility of lipophilic IPT, eliminating the dissolution rate-limiting step that restricts its oral absorption [25].

Liquid lipid components are critical determinants for the structural stability, delivery efficiency, and biosafety of NLC delivery systems [26]. The selection of natural, biocompatible, and bioactive liquid lipids is essential to endow lipid carriers with dual advantages of safe delivery and synergistic efficacy, which is highly consistent with the development concept of modern green oral preparations and food–medicine integrated formulations. As a natural plant-derived lipid with typical food–medicine homology characteristics, grape seed oil (GSO) is widely applied in the food, healthcare, and pharmaceutical industries due to its excellent biocompatibility, biodegradability, and non-toxicity, making it an ideal liquid lipid candidate for NLC construction [27]. Regarding formulation performance, GSO exhibits suitable lipophilicity and fluidity, enabling it to rectify the imperfect crystal structure within NLCs. This not only enhances the system’s drug loading capacity and colloidal stability, but also facilitates carrier penetration across the intestinal mucosal barrier, thereby improving the oral absorption of the encapsulated IPT [28]. With respect to biological activity, GSO is rich in unsaturated fatty acids, which possess notable antioxidant and anti-aging properties [29]. Moreover, GSO can scavenge excess intracellular free radicals, thereby alleviating oxidative damage to cells, and it also modulates the activity of oxidative stress-related enzymes [30]. Importantly, GSO can exert synergistic antioxidant and anti-aging effects with IPT. This perfect combination achieves the innovative integration of food–medicine homology and excipient-drug synergism, realizing safe carrier delivery while amplifying the intrinsic pharmacological and healthcare efficacy of IPT.

Glyceryl monostearate (GMS) was chosen as the solid lipid skeleton owing to its food-grade property, appropriate melting characteristic, and high compatibility with lipophilic IPT [31]. However, single GMS forms dense ordered crystals that cause drug leakage [32]. So, GSO was incorporated as a liquid lipid to disrupt the perfect crystal arrangement of GMS, creating loose internal cavities. Thus, the introduction of GSO can compensate for the shortcomings of GMS applications.

Benefiting from the green formulation composition and unique structural superiority, GSO-modified NLC delivery systems exhibit great practical application value and industrialization potential. This lipid delivery platform can be fabricated via simple, controllable and organic solvent-free preparation processes, which conforms to the green and safe manufacturing requirements of oral pharmaceuticals and functional foods [33]. With excellent formulation adjustability, the system can be flexibly optimized according to the physicochemical properties of different bioactive components and diverse application scenarios [34]. Moreover, it is highly compatible with mature industrial technologies, including high-pressure homogenization, spray drying, and sterilization, providing reliable technical support for large-scale industrial production [35,36]. Collectively, such NLCs can fundamentally address the inherent defects of IPT, including poor water solubility, insufficient intestinal absorption, and low oral bioavailability, thereby greatly expanding the application prospects of IPT in functional foods, health products, and clinical oral formulations.

Therefore, the present study successfully fabricated IPT-loaded nanostructured lipid carriers (IPT@NLCs) using GMS as a solid lipid and natural GSO as a liquid lipid. The basic physicochemical properties of IPT@NLCs, including particle size, polydispersity index (PDI), zeta potential, microscopic morphology, and in vitro release characteristics were systematically characterized, and their drug loading capacity and encapsulation efficiency were quantitatively evaluated. Meanwhile, a Caco-2/HT29-MTX-E12 co-culture cell model was established to investigate the cellular uptake and transmembrane transport behavior of IPT@NLCs, so as to clarify its intestinal absorption mechanism. The 3T3-L1 cell oxidative damage model was further applied to verify the synergistic antioxidant activity of the optimized delivery system. In addition, in vivo pharmacokinetic experiments in rats were carried out to establish a quantitative detection method for plasma IPT content and evaluate the in vivo bioavailability improvement effect. Combined with GI safety evaluation and intestinal distribution analysis, this study comprehensively explores the potential application value of IPT@NLCs as a high-efficiency oral delivery system for functional food and pharmaceutical development.

## 2. Results and Discussion

### 2.1. Characteristics of NLCs

**Figure 1:**
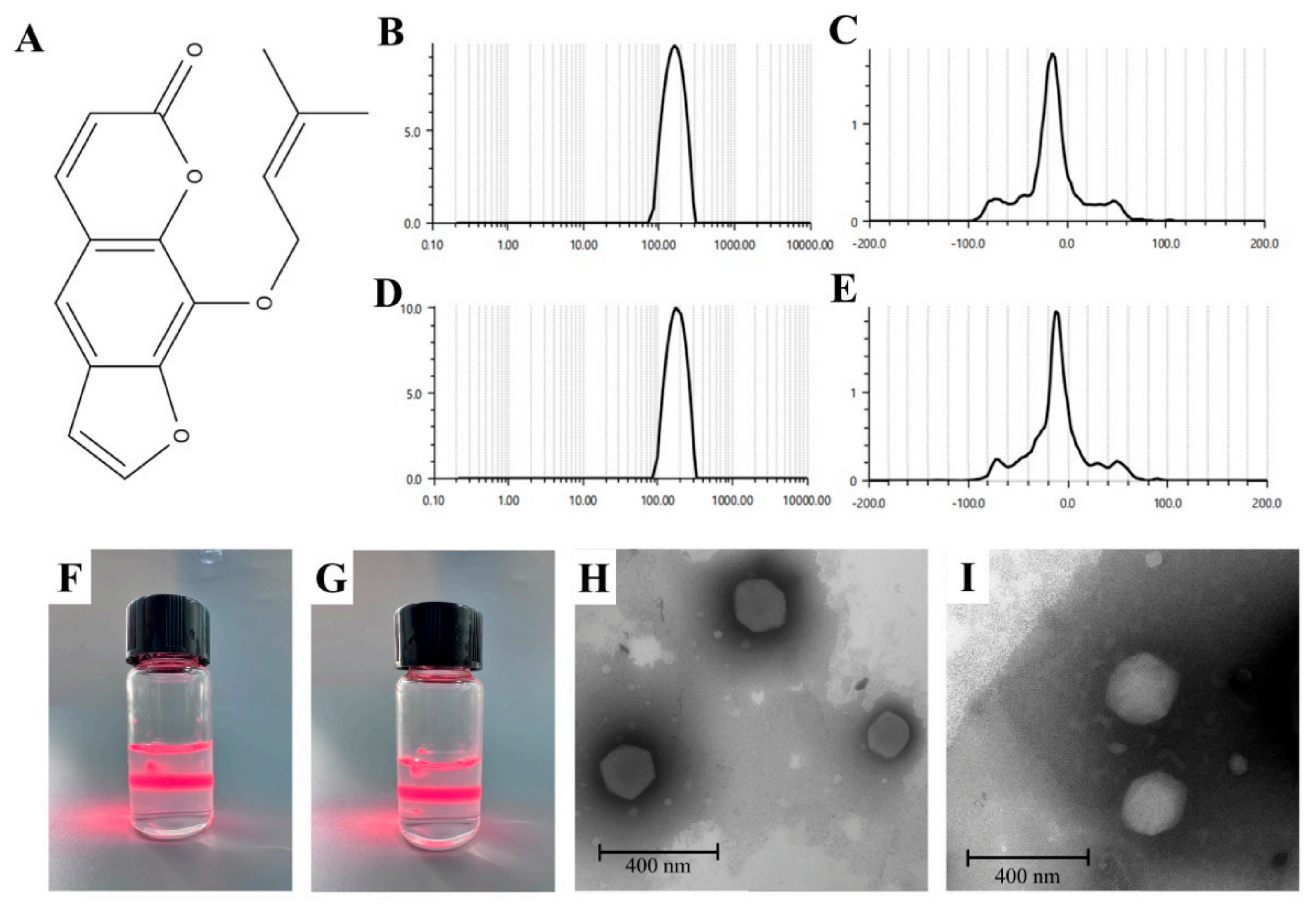
Characterization of NLCs. (**A**) The chemical structure of IPT. (**B**) Particle size distribution of Blank NLCs. (**C**) Zeta potential of Blank NLCs. (**D**) Particle size distribution of IPT@NLCs. (**E**) Zeta potential of IPT@NLCs. (**F**) Appearance of Blank NLCs. (**G**) Appearance of IPT@NLCs. (**H**) TEM images of Blank NLCs. (**I**) TEM images of IPT@NLCs.

As shown in Figure 1B,D and Table 1, the particle size of Blank NLCs was 162.72 ± 2.38 nm with a PDI of 0.174 ± 0.015; in contrast, IPT@NLCs exhibited a mean particle size of 186.63 ± 1.65 nm and a PDI of 0.188 ± 0.008. The PDI values for both Blank NLCs and IPT@NLCs remained below 0.3, indicating a narrow and homogeneous particle size distribution across all prepared formulations. As presented in Figure 1C,E and Table 1, the zeta potential of Blank NLCs was −21.40 ± 0.10 mV, whereas that of IPT@NLCs was −18.60 ± 0.30 mV, indicating good colloidal stability of the formulation system. Visually, all NLCs samples appeared as milky-white, homogeneous liquids. Upon dilution, as illustrated in Figure 1F,G, all formulations exhibited weak opalescence and a pronounced Tyndall effect. TEM images revealed that both Blank NLCs and IPT@NLCs detected by negative staining had a relatively regular spherical morphology and a relatively uniform distribution (Figure 1H,I).

### 2.2. Encapsulation Efficiency and Drug Loading

Encapsulation efficiency (EE) and drug loading (DL) are key indicators for assessing the quality of NLCs. EE is mainly governed by the physicochemical properties of the loaded compounds as well as intermolecular interactions between drugs and carriers [37]. By contrast, DL is affected by the miscibility of active ingredients in molten lipids, lipid polymorphism, and the internal structure of NLCs [38].

As summarized in Table 1, the DL and EE of IPT@NLCs were 9.08 ± 0.23% and 99.54 ± 0.10%, respectively. These data demonstrate that NLCs possess excellent encapsulation capability and high drug loading capacity. Benefiting from this superior encapsulation performance, the high EE can effectively protect IPT from degradation in the acidic gastric environment and enable the loaded IPT to efficiently penetrate the biological mucus barrier. The excellent EE value reflects the high compatibility and strong binding affinity between IPT and the lipid matrix of NLCs. The incorporation of GSO constructs a semi-solid lipid network with internal cavities, which further elevates the DL of NLCs and improves the drug loading efficiency and physicochemical stability of the formulations [39].

The ultra-high encapsulation efficiency of IPT@NLCs endows the nanocarriers with multiple additional superior properties. First, high EE substantially reduces the amount of unencapsulated Free IPT, preventing the precipitation of lipophilic free drug within gastrointestinal fluids. Second, it eliminates the pronounced initial burst release originating from surface-adsorbed Free IPT, facilitating stable sustained drug delivery for up to 48 h in accordance with Higuchi diffusion-controlled release kinetics. Lastly, the extremely high encapsulation efficiency reduces the metabolic degradation of IPT before intestinal absorption, which provides critical conditions for elevating the oral bioavailability.

### 2.3. In Vitro Release

**Figure 2:**
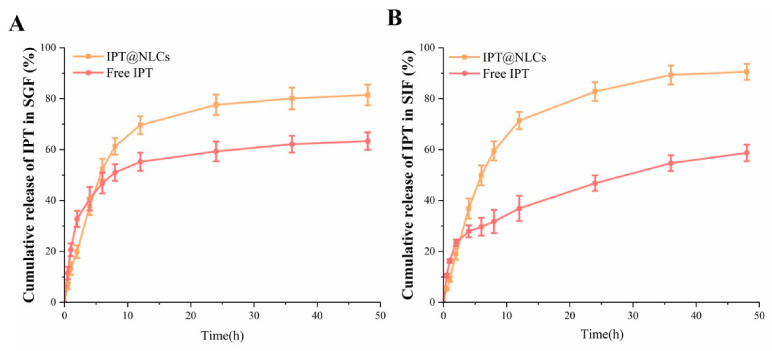
In vitro release. (**A**) In vitro release profiles of Free IPT and IPT@NLCs in SGF (mean ± SD, n = 3). (**B**) In vitro release profiles of Free IPT and IPT@NLCs in SIF (mean ± SD, n = 3).

In vitro release profiles of Free IPT and IPT@NLCs were evaluated in simulated gastric fluid (SGF) and simulated intestinal fluid (SIF). In SGF (Figure 2A), IPT@NLCs exhibited a lower release rate than Free IPT during the initial 4 h, indicating a relatively slower early-stage release under gastric conditions. With prolonged incubation, however, the cumulative release of IPT from IPT@NLCs gradually increased and became higher than that of Free IPT, reaching 81.49 ± 4.08% at 48 h, whereas Free IPT plateaued at approximately 63.35 ± 4.05%. In SIF (Figure 2B), IPT@NLCs showed minimal drug release within the first 2 h, suggesting limited premature leakage of IPT from the nanocarriers. After 4 h, the cumulative release increased markedly, accompanied by an accelerated release rate. Over the 48 h period, the cumulative release of IPT from IPT@NLCs reached 90.56 ± 3.12%, which was substantially higher than that of Free IPT at 58.74 ± 3.25%. These results demonstrate that IPT@NLCs achieved a higher cumulative release than Free IPT in both simulated gastrointestinal media, particularly under intestinal conditions.

To further clarify the release behavior of IPT in different simulated gastrointestinal media, the in vitro release profiles were fitted using zero-order, Higuchi, and Korsmeyer–Peppas kinetic models [40,41]. As shown in Table 2, the zero-order model showed relatively low correlation coefficients for both Free IPT and IPT@NLCs in SGF and SIF, with R^2^ values ranging from 0.5558 to 0.8078, indicating that IPT release did not follow a constant-rate pattern. In contrast, the Higuchi model provided a better fit for all groups, with R^2^ values above 0.9551, suggesting that diffusion played an important role in the release process. Notably, the Korsmeyer–Peppas model exhibited the highest fitting accuracy for IPT@NLCs in both SGF and SIF, with R^2^ values of 0.9945 and 0.9959, respectively, indicating that this model more appropriately described the release behavior of IPT from the NLC system.

The release exponent n further supported differences in the release mechanisms between Free IPT and IPT@NLCs. For Free IPT, the n values were 0.4174 in SGF and 0.3492 in SIF, suggesting a predominantly Fickian diffusion-controlled release pattern, particularly in SIF. By contrast, IPT@NLCs showed higher n values of 0.8042 in SGF and 0.9047 in SIF, implying that IPT release from NLCs involved a more complex mechanism rather than simple diffusion alone [42]. Specifically, the n value of IPT@NLCs in SGF fell within the range of anomalous non-Fickian transport, suggesting that the release process was jointly regulated by drug diffusion through the lipid matrix and relaxation or erosion of the nanocarriers. In SIF, the n value exceeded 0.89, indicating a stronger contribution from carrier matrix relaxation, erosion, or structural rearrangement, accompanied by diffusion, to the release of IPT from NLCs.

Overall, these kinetic fitting results indicate that IPT release from NLCs was best described by the Korsmeyer–Peppas model, while the good fit of the Higuchi model further suggests that diffusion-associated processes contributed substantially to the release behavior in both simulated gastric and intestinal fluids.

### 2.4. Cellular Uptake

To realistically simulate the intestinal mucus barrier and assess mucus-associated cellular uptake of NLCs, we used a Caco-2/HT29-MTX-E12 co-culture model [43]. Because Caco-2 monolayers are devoid of goblet cells and do not secrete mucus, they fall short of fully recapitulating the native intestinal microenvironment. This limitation can be circumvented by co-culturing with HT29-MTX-E12 cells, which differentiate into mucus-producing goblet cells and reconstitute an epithelial–mucus barrier that is functional for in vitro transport studies [44]. Given that intestinal mucus forms a protective gel that can entrap exogenous particulates, we designed cellular uptake experiments under two conditions—one with an intact mucus layer and the other with mucus removed by N-acetylcysteine (NAC) pretreatment—so as to specifically evaluate the influence of mucus on nanoparticle internalization [45].

**Figure 3:**
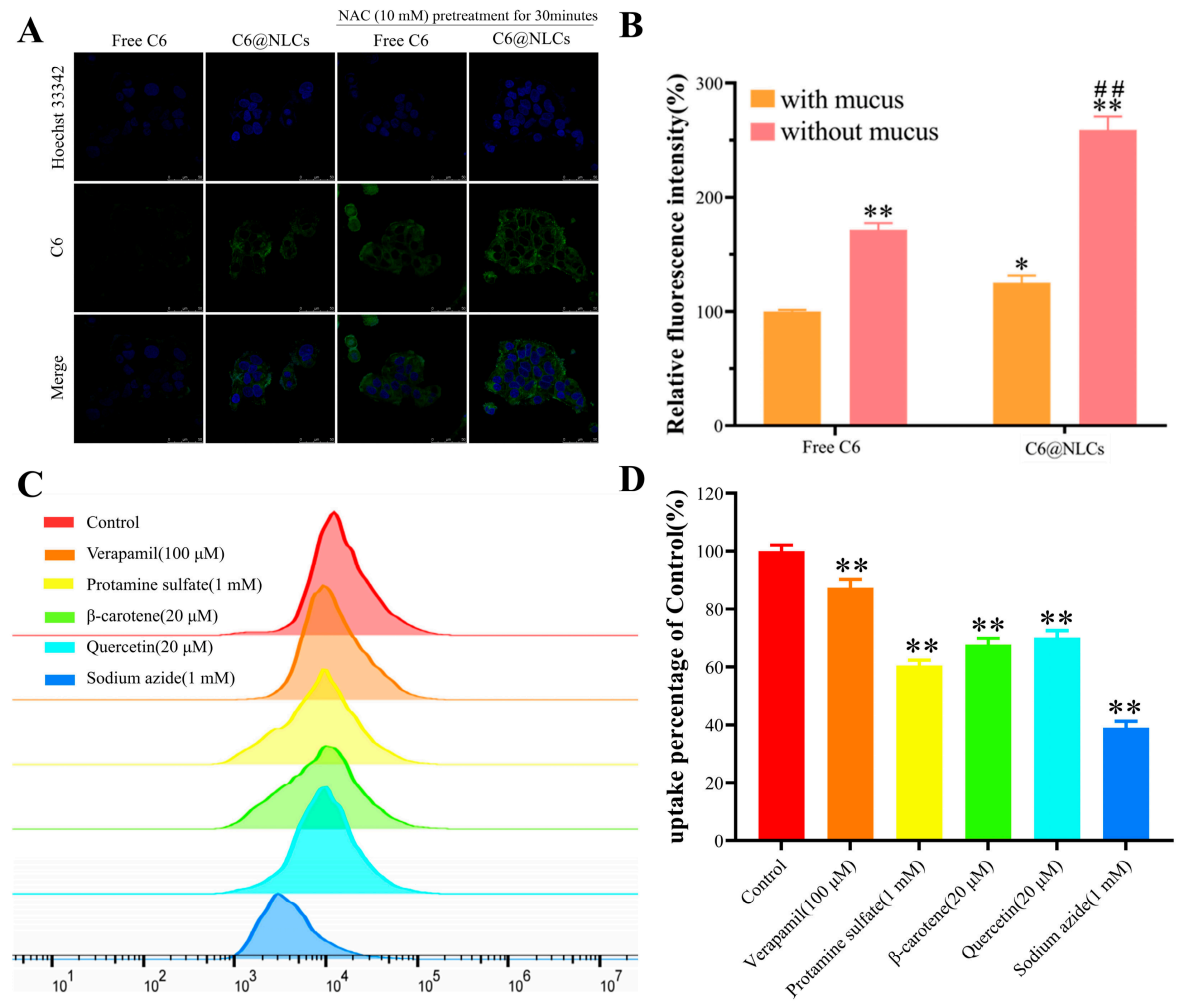
Cellular uptake and uptake mechanisms. (**A**) Uptake in Caco-2/HT29-MTX-E12 co-cultured cells. (**B**) Relative fluorescence quantification of cellular uptake. * *p* < 0.05, ** *p* < 0.01 vs. Free C6 with mucus. ^##^ *p* < 0.01 vs. Free C6 without mucus. (**C**) The chromatogram of C6@NLCs uptake by co-cultured cells. (**D**) Effects of added substances on cellular uptake. ** *p* < 0.01 vs. the Control.

Cellular uptake among the different groups was compared via fluorescence imaging and quantified with ImageJ 1.54 (Figure 3A,B). In these images, green fluorescence corresponds to C6-labeled nanoparticles, whereas blue fluorescence marks Hoechst 33342-stained nuclei. Quantification revealed that, irrespective of whether mucus was present, C6@NLCs were taken up by epithelial cells to a significantly greater extent than Free C6. Although the mucus layer did impose a modest barrier that marginally reduced nanoparticle uptake, the NLC formulation still outperformed Free C6 by a substantial margin in terms of epithelial cell internalization.

The superior mucus-penetrating capability of NLCs can be attributed to their lipid-based architecture. With liquid lipid cores, these nanocarriers are highly deformable, enabling them to navigate the porous mucin network while reducing non-specific hydrophobic interactions with mucus components [46]. In addition, Solutol^®^ HS 15—an amphiphilic non-ionic surfactant—diminishes electrostatic adhesion between the nanoparticles and negatively charged mucin, thereby enhancing particle mobility within the mucus layer and accelerating their passage across this barrier [47]. This enhances particle mobility within the mucus layer and accelerates penetration toward underlying intestinal epithelial cells.

### 2.5. Uptake Mechanisms

Verapamil is a classic P-glycoprotein (P-gp) inhibitor [48]. Protamine sulfate inhibits cellular uptake by blocking adsorption-mediated endocytosis [49]. β-Carotene enhances intestinal epithelial barrier integrity by upregulating tight junction protein expression, thereby indirectly restricting nanoparticle transmembrane internalization [50]. Quercetin inhibits clathrin- and caveolae-independent endocytic pathways [51]. Sodium azide blocks energy-dependent transport by depleting intracellular ATP, thus suppressing all ATP-consuming endocytic processes [52]. Figure 3C,D show the relative cellular uptake of C6@NLCs after 1 h of pre-incubation with each pathway inhibitor, followed by 2 h of co-incubation with nanoparticles. Compared with the untreated Control, sodium azide and protamine sulfate caused the greatest reduction in uptake. Uptake in the sodium azide group was only 39.01% of the Control value, while that in the protamine sulfate group was 60.42% of the Control. Other inhibitors also reduced nanoparticle internalization to varying but consistently observable extents. The strong suppression by sodium azide and protamine sulfate shows that NLC internalization depends mainly on energy-dependent active transport and adsorption-mediated endocytosis. P-gp efflux, caveolae-independent endocytosis, and tight junction barrier permeability collectively act as auxiliary regulatory factors that influence the overall cellular uptake efficiency of NLCs.

### 2.6. Transport Study

**Figure 4:**
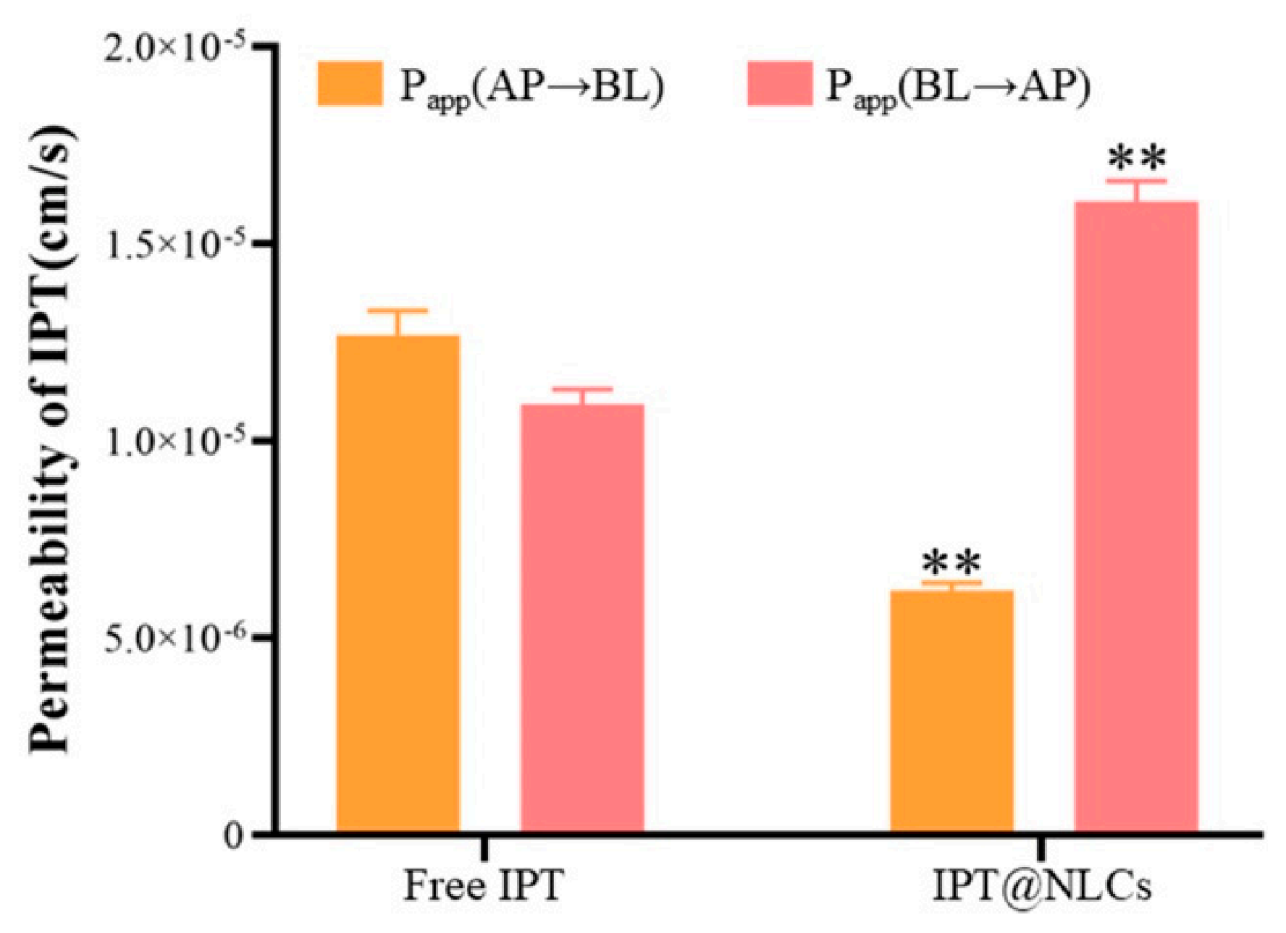
AP → BL and BL → AP permeability of Free IPT and IPT@NLCs. ** *p* < 0.01 vs. Free IPT.

The apparent permeability coefficient (P_app_) is a core quantitative parameter to assess the transmembrane absorption capacity of drugs across the intestinal epithelial barrier [53]. The bidirectional transport profiles (absorption direction: apical-to-basolateral, AP → BL; secretory efflux direction: basolateral-to-apical, BL → AP) of Free IPT and IPT@NLCs across the Caco-2/HT29-MTX-E12 co-culture monolayer are summarized in Figure 4 and Table 3. The efflux ratio, calculated as P_app_ (BL → AP)/P_app_ (AP → BL), serves as a standard criterion to distinguish dominant intestinal transport mechanisms. According to the calculated data in Table 3, Free IPT exhibited an efflux ratio of 0.861 close to unity, confirming that the transmembrane transport of unencapsulated IPT relies almost entirely on passive diffusion. In contrast, IPT@NLCs displayed an efflux ratio exceeding the commonly accepted threshold of 1.5, strongly suggesting that NLC-mediated encapsulation shifts the dominant transport mechanism from passive diffusion to energy-dependent active transport [54]. The bidirectional transport data suggest that nanoencapsulation alters the epithelial transport behavior of IPT compared with the free drug. Free IPT showed a slightly higher AP → BL P_app_ than IPT@NLCs, which is consistent with passive diffusion of a dissolved small molecule across the epithelial monolayer. In contrast, for nanoparticle formulations, the amount of drug detected in the receiver chamber over a limited in vitro period may not fully reflect total cellular uptake or transcellular processing because intracellular retention and delayed drug release can all influence the apparent P_app_ values. Therefore, these transport results are more indicative of altered transport behavior than of a simple increase in absorptive permeability. The improved oral absorption of IPT@NLCs is better supported by the combined evidence of enhanced cellular uptake, stronger intestinal distribution, and markedly increased in vivo oral bioavailability.

### 2.7. H_2_O_2_-Induced Oxidative Stress

**Figure 5:**
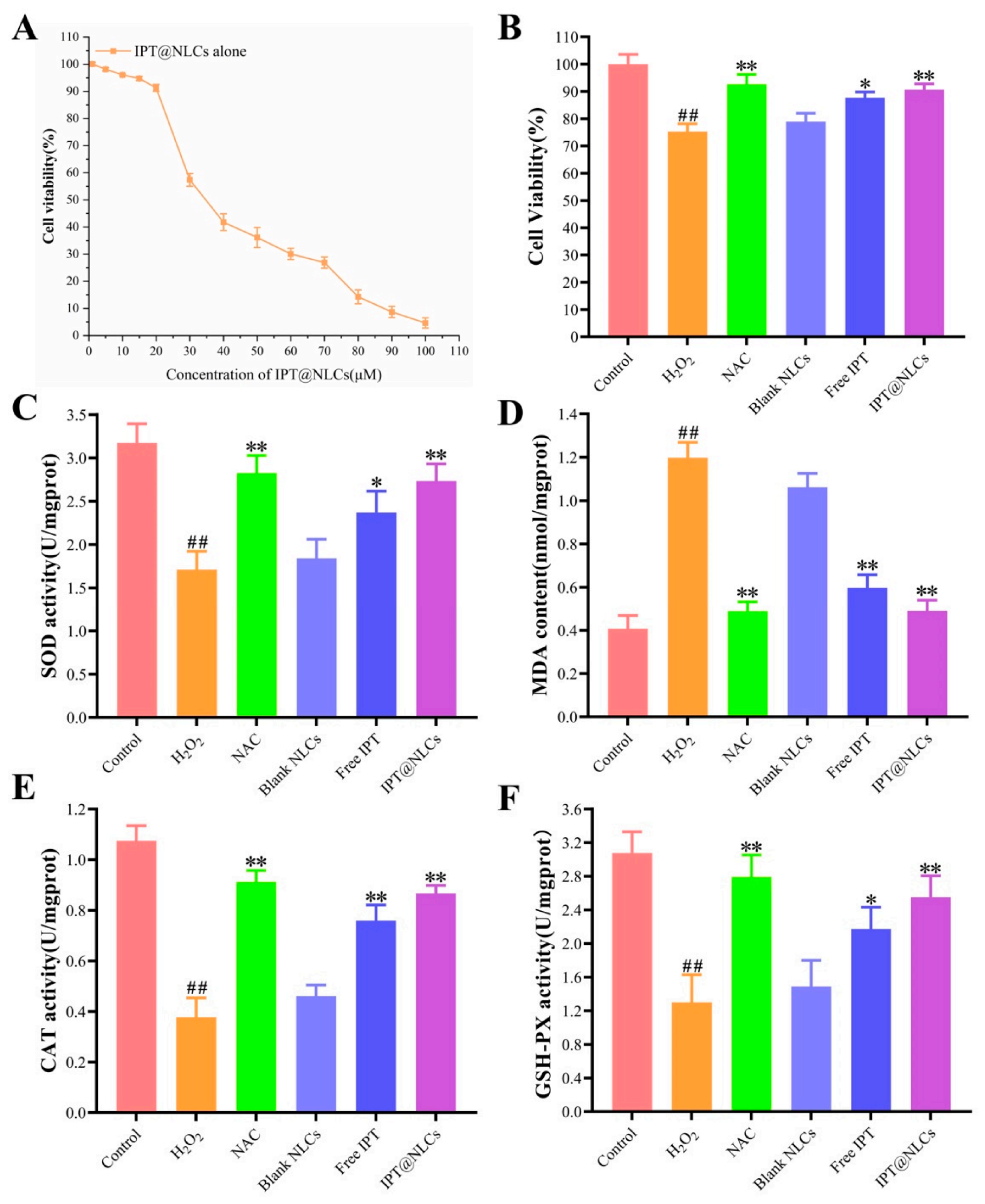
Oxidative stress in 3T3-L1 cells. (**A**) Cell growth curve after administration of IPT@NLCs. (**B**) Cell viability of H_2_O_2_-stimulated 3T3-L1 adipocytes after different treatments. ^##^ *p* < 0.01 vs. the Control. * *p* < 0.05, ** *p* < 0.01 vs. H_2_O_2_. (**C**) SOD activity. ^##^ *p* < 0.01 vs. the Control. * *p* < 0.05, ** *p* < 0.01 vs. H_2_O_2_. (**D**) MDA content. ^##^ *p* < 0.01 vs. the Control. ** *p* < 0.01 vs. H_2_O_2_. (**E**) CAT activity. ^##^ *p* < 0.01 vs. the Control. ** *p* < 0.01 vs. H_2_O_2_. (**F**) GSH-Px activity. ^##^ *p* < 0.01 vs. the Control. * *p* < 0.05, ** *p* < 0.01 vs. H_2_O_2_ (mean ± SD, n = 3).

Long-term excess energy intake breaks metabolic homeostasis and elevates oxidative stress, which accelerates cellular senescence and promotes adipogenesis, ultimately causing obesity [55,56,57]. The 3T3-L1 preadipocyte model is widely used to evaluate lipid metabolism and natural compounds with antioxidant, anti-aging, and anti-obesity effects [58]. As a natural coumarin, IPT exerts potent antioxidant and anti-senescence effects by alleviating oxidative stress. The cytotoxicity profile of IPT@NLCs toward 3T3-L1 cells after 24 h incubation is presented in Figure 5A. At 15 μM IPT equivalent concentration, the cell viability remained above 95%, while higher concentrations induced a gradual drop in cell survival. Accordingly, 15 μM IPT was chosen for the follow-up oxidative damage intervention experiments. Oxidative stress imposes multiple detrimental impacts on cells, including accelerated senescence and suppressed proliferative potential. In our viability assay, cellular viability fell to roughly 75% after 24 h of incubation with 300 μM H_2_O_2_, validating the successful construction of an in vitro oxidative stress cell model. Treatment with the positive antioxidant NAC significantly restored cell viability under oxidative damage (*p* < 0.01). Blank NLCs exerted no obvious protective effect against H_2_O_2_-induced cell injury. By contrast, Free IPT moderately elevated cell viability (*p* < 0.05), while IPT@NLCs exhibited a more prominent cytoprotective capacity and markedly rescued the loss of cell viability caused by oxidative stress (*p* < 0.01) (Figure 5B).

### 2.8. Determination of Oxidative Stress Level

Intracellular total SOD, CAT, GSH-Px activities, and MDA levels were measured to evaluate the protective effect of IPT@NLCs against H_2_O_2_-induced oxidative stress in 3T3-L1 adipocytes (Figure 5C–F). Compared with the Control group, H_2_O_2_ treatment drastically suppressed the activities of SOD, CAT, and GSH-Px while markedly elevating MDA contents (*p* < 0.01), which confirmed severe intracellular oxidative injury. Blank NLCs incorporated solely with GSO only slightly recovered antioxidant enzyme activities and exerted negligible inhibitory effects on MDA generation, revealing weak inherent antioxidant activity of the blank lipid nanocarriers. Free IPT significantly restored the activities of antioxidant enzymes (*p* < 0.05 or *p* < 0.01) and mitigated lipid peroxidation. By contrast, IPT@NLCs exhibited robust antioxidant capacity, which profoundly upregulated SOD, CAT, and GSH-Px and remarkably reduced MDA accumulation (*p* < 0.01). The outstanding antioxidant performance of IPT@NLCs could be attributed to the synergistic antioxidative interaction between encapsulated IPT and GSO within the nanolipid matrix. Collectively, H_2_O_2_ triggered severe oxidative stress characterized by depleted antioxidant enzymes and massive MDA deposition. GSO-loaded Blank NLCs merely provided marginal antioxidative protection, Free IPT exerted moderate alleviation against oxidative damage, and the IPT@NLC nanoformulation achieved potent antioxidant and anti-lipid peroxidation effects through the synergistic action of IPT and GSO.

Collectively, our experimental data revealed that IPT treatment lowered MDA accumulation in oxidatively injured cells while simultaneously upregulating the activities of three core antioxidant enzymes: SOD, CAT and GSH-Px. These findings demonstrate that IPT exerts potent antioxidant effects by reinforcing the activity of endogenous antioxidant enzymes, thereby strengthening cellular antioxidant resistance against oxidative damage.

### 2.9. Pharmacokinetic Study

**Figure 6:**
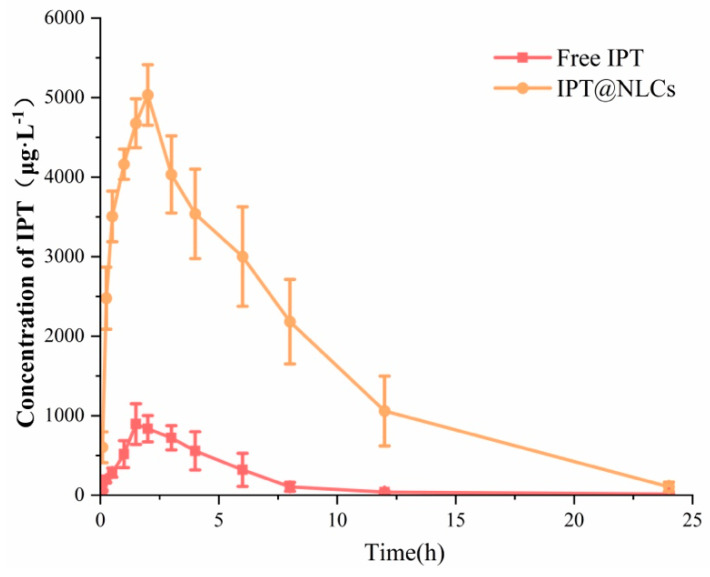
Mean plasma concentration–time profiles of Free IPT and IPT@NLCs (mean ± SD, n = 5).

Plasma drug concentration data of Free IPT and IPT@NLCs were imported into DAS 2.0 pharmacokinetic software to generate drug concentration–time curves and calculate relevant pharmacokinetic parameters, as summarized in Figure 6 and Table 4. According to the data listed in Table 4, oral gavage administration of IPT@NLCs drastically elevated the peak plasma C_max_ of IPT in rats relative to Free IPT. Specifically, the C_max_ value rose from 943.37 ± 196.88 μg·L^−1^ to 5101.45 ± 321.48 μg·L^−1^ after nanoformulation. The T_max_ was prolonged from 1.60 ± 0.22 h to 1.90 ± 0.22 h, and the MRT_(0-t)_ and MRT_(0-∞)_ were extended accordingly. The AUC_(0-t)_ values of Free IPT and IPT@NLCs were 4396.21 ± 1136.77 μg·L^−1^·h and 41091.61 ± 7649.75 μg·L^−1^·h, respectively. The bioavailability of IPT in the IPT@NLC group was 934.74% of that in the Free IPT group. The C_max_, AUC_(0-t)_, AUC_(0-∞)_, and MRT_(0-t)_ values all had statistical significance (*p* < 0.01). These results indicate that NLCs can effectively promote the oral absorption of IPT and improve its relative bioavailability.

### 2.10. Gastrointestinal Safety Assay

**Figure 7:**
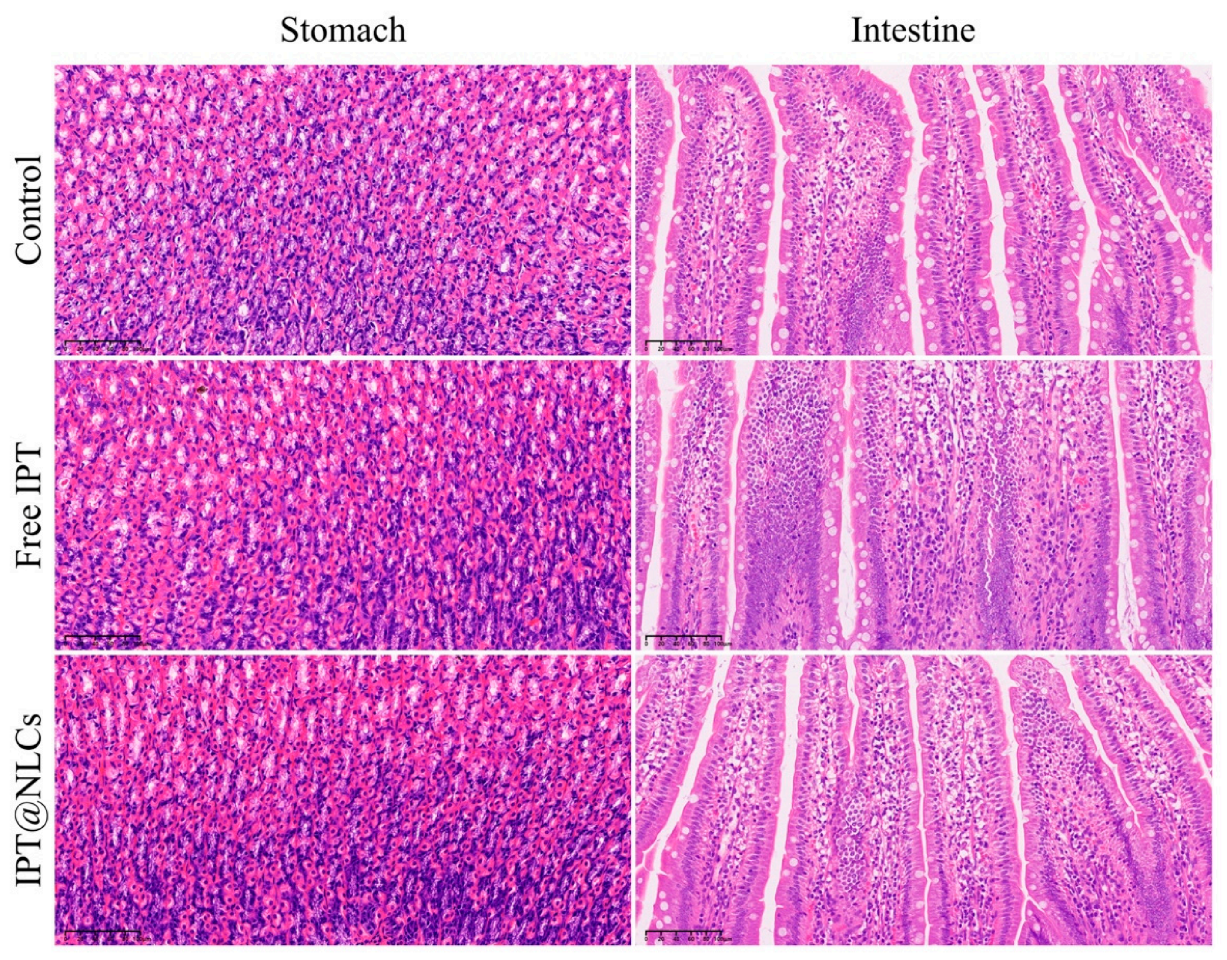
Gastrointestinal safety assessment by H&E staining.

An ideal oral drug carrier should be able to maintain or enhance the gastrointestinal safety of drugs. In this study, the safety of NLCs as a drug carrier was evaluated through gastrointestinal irritation tests. As shown in Figure 7, compared with the Control group, the Free IPT group showed no mild keratinization on the gastric wall surface, no mild congestion or cellular atypia in the intestinal lamina propria, and no abnormal changes in intestinal villi, which indicates that IPT has good gastrointestinal safety. Notably, the IPT@NLC treatment group displayed identical normal histological features of stomach and intestinal tissues. No inflammatory lesions, mucosal erosion, cell necrosis, or villous atrophy were observed in gastrointestinal sections after repeated oral administration of IPT-loaded nanoparticles. This finding clearly demonstrates that the NLC matrix composition does not exacerbate gastrointestinal mucosal irritation induced by IPT, supporting its potential as a novel oral drug delivery system.

### 2.11. Intestinal Distribution Studies

**Figure 8:**
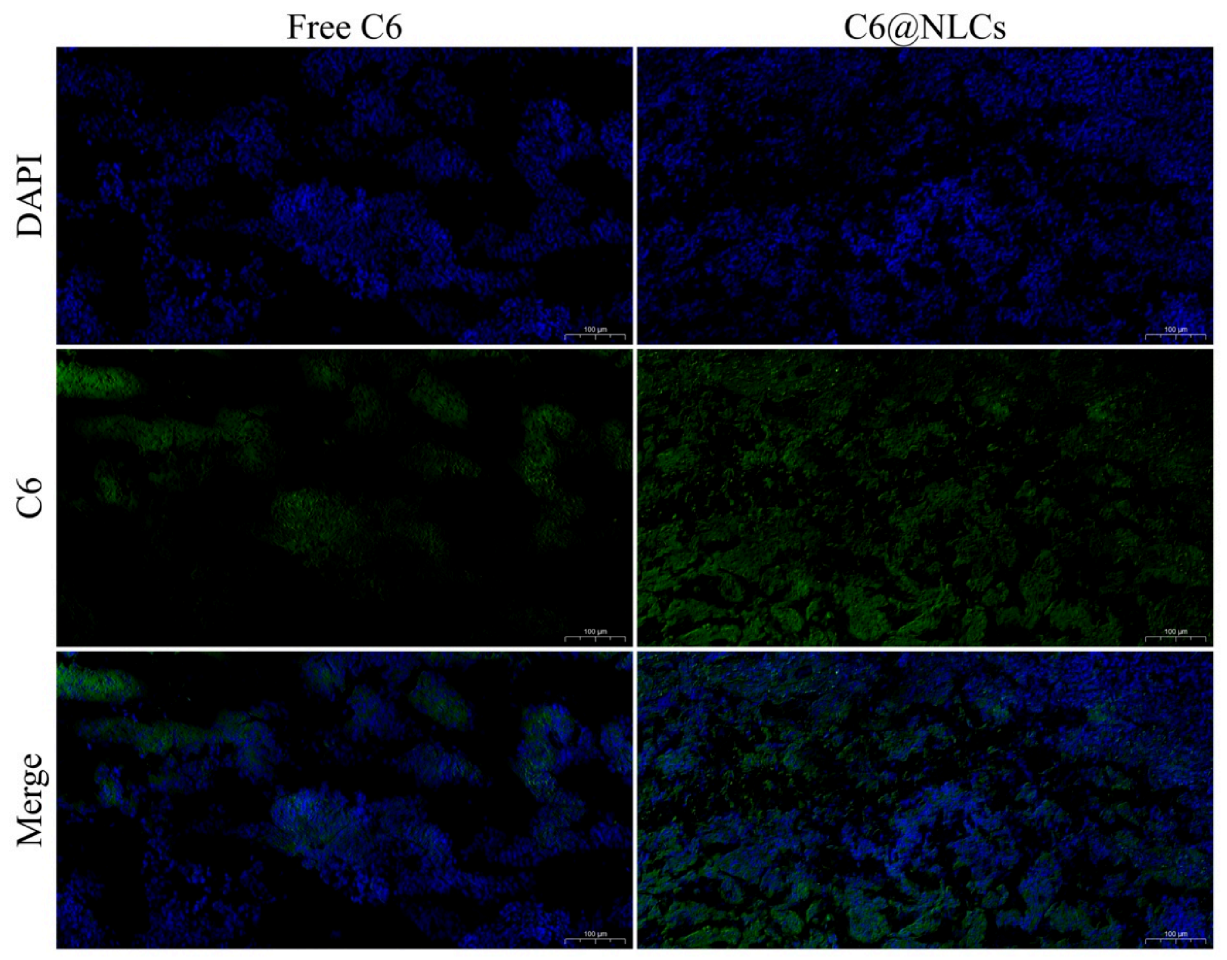
CLSM imagery of the biodistribution in the intestine.

To observe the distribution of NLCs in the intestine, in this study, C6 was used as a fluorescent probe to replace IPT, and Free C6 and C6@NLCs were prepared. Figure 8 presents CLSM images: blue fluorescence marks the intestinal cell nuclei, whereas green fluorescence indicates C6. A clear green signal is observed in the intestinal segments, confirming that C6 is taken up by the intestinal tissue. Compared with the Free C6 group, the C6@NLC group displays a markedly stronger green signal, suggesting that NLCs facilitate greater intestinal absorption. This result demonstrates that NLCs can significantly improve the intestinal uptake efficiency of C6.

## 3. Materials and Methods

### 3.1. Materials

IPT (>98% purity) was supplied by Kasaisi pharmaceutical technology Co., Ltd. (Nanjing, China). Osthole, GMS, and GSO were purchased from McLean Biochemical Technology Co., Ltd. (Shanghai, China). The Caco-2 cell line was purchased from Fuheng Biotechnology Co., Ltd. (Shanghai, China). The HT29-MTX-E12 cell line was purchased from Mcellbank (Shanghai, China). The 3T3-L1 cell line was purchased from Servicebio Co., Ltd. (Wuhan, China). All remaining chemicals were either of analytical grade or chromatographic grade and were utilized in their original state.

### 3.2. Animals

Male Sprague–Dawley (SD) rats weighing 200 ± 20 g were sourced from Hangzhou Ziyuan Laboratory Animal Technology Co., Ltd., Hangzhou, China (Animal Production License: SCXK (Zhejiang) 2024-0004) and maintained at the Animal Experiment Center of Wannan Medical University. Prior to testing, all animals underwent a 7-day acclimatization period with unrestricted access to feed and water. The housing facility was kept at a constant temperature of 25 ± 5 °C and a relative humidity of 45 ± 5%.

### 3.3. In Vitro Analysis Methods for IPT

All in vitro samples were quantified via an Agilent 1290 UPLC platform (Agilent Technologies, Santa Clara, CA, USA). We set the UV detection wavelength for IPT to 300 nm, with each sample injection volume controlled at 10 μL. A Boltimate-C_18_ column (2.1 × 100 mm, 2.7 μm) served as the separation medium. The column oven remained stable at 30 °C alongside a steady flow rate of 0.3 mL·min^−1^. Water (eluent A) and methanol (eluent B) formed the binary mobile phase, and the linear gradient elution schedule was arranged as follows: 55% A from 0 to 1.5 min; 55–70% A from 1.5 to 5.5 min; 70–80% A from 5.5 to 6.5 min; 80–88% A from 6.5 to 6.6 min; and 88% A from 6.6 to 10 min. The linear regression equation of IPT was A = 59.32C − 6.0112 (R = 0.9997). This method showed a linear relationship in the range of 0.05–200.00 μg·mL^−1^.

### 3.4. Preparation of Investigated Formulations

#### 3.4.1. Preparation of Blank NLCs

GMS (60 mg) and GSO (90 mg) were combined at a weight ratio of 1:1.5, and then fully melted and magnetically stirred at 75 °C for 30 min to prepare the oil phase. Separately, 300 μL Solutol^®^ HS 15 was dissolved in deionized water and agitated at 90 °C for the same 30 min to create the aqueous fraction. After heating both phases to preset temperatures, we blended the aqueous fraction into the warm oil phase and mixed the system at 75 °C for an extra 30 min to form homogeneous precursors. This crude mixture underwent high-pressure homogenization at 5000 bar for 10 min to produce Blank NLCs.

#### 3.4.2. IPT@NLCs of the Build

To synthesize IPT@NLCs, GMS (60 mg) and GSO (90 mg) were combined at a weight ratio of 1:1.5, followed by the addition of 16 mg of precisely weighed IPT. The blend was fully melted and agitated at 75 °C for 30 min to serve as the lipid oil phase. Separately, 300 μL Solutol^®^ HS 15 was dissolved in deionized water under stirring at 90 °C for 30 min to prepare the aqueous fraction. After both phases reached the target temperature, we slowly blended the aqueous fraction into molten oil and maintained agitation at 75 °C for another 30 min to form homogeneous pre-emulsion. The resulting crude suspension underwent high-pressure homogenization at 5000 bar over 10 min to generate IPT@NLCs.

### 3.5. Characterization of the NLCs

#### 3.5.1. Particle Size, PDI, and Zeta Potential

The particle size distribution and zeta potential of the Blank NLCs and IPT@NLCs were characterized by a Litesizer 500 analyzer (Anton Paar, Graz, Austria). Freshly prepared Blank NLCs and IPT@NLCs were diluted with ultrapure water, and then placed into particle size cuvettes and potential cuvettes for determination. Each sample was measured in triplicate, and the average particle size, PDI, and zeta potential were recorded.

#### 3.5.2. Observation of Appearance

The prepared Blank NLCs and IPT@NLCs were transferred into transparent glass vials. A red laser pointer was used to irradiate the sample vials vertically, and the Tyndall effect was observed to preliminarily determine the formation status. The observation results were recorded and photographs were taken.

#### 3.5.3. Transmission Electron Microscopy

Transmission electron microscopy (TEM; JEOL, Tokyo, Japan) was used to visualize the morphology of the Blank NLCs and IPT@NLCs. After diluting each sample with ultrapure water and vortexing thoroughly, we transferred a tiny droplet of the suspension onto a 230-mesh carbon-coated copper grid. The grids were air-dried at ambient temperature and then negatively stained with 2% phosphotungstic acid. After full dehydration, TEM observation was carried out at different magnifications.

#### 3.5.4. Encapsulation Efficiency and Drug Loading

The EE of IPT@NLCs was measured using the ultrafiltration centrifugation method described in Reference [59]. IPT@NLCs were loaded into a 3 kDa ultrafiltration centrifuge tube and spun at 12,000 rpm for 15 min. We collected the filtrate and performed UPLC quantification to detect Free IPT concentration (W_F_). For parallel total drug testing, IPT@NLCs were blended with methanol at a 1:9 volume ratio, followed by 10 min of sonication (250 W, 40 kHz) to break down the formulations. After another 15 min of centrifugation at 12,000 rpm, the supernatant was passed through a 0.22 μm microporous membrane, and UPLC testing was implemented to quantify the total IPT concentration (W_T_). The encapsulation efficiency and drug loading values of IPT were calculated via Equations (1) and (2):

(1) $$EE\%=\frac{W_{T}{-W}_{F}}{W_{T}}\times 100\%$$

(2) $$DL\%=\frac{W_{T}{-W}_{F}}{W_{M}}\times 100\%$$

where *W_T_* is the total mass of IPT in the IPT@NLCs system, *W_F_* is the mass of unencapsulated Free IPT in the filtrate, and *W_M_* refers to the total mass of the IPT@NLCs formulation.

#### 3.5.5. In Vitro Release

We adopted the dialysis bag method to characterize the in vitro drug release profile of IPT@NLCs. Two test formulations, IPT@NLCs and pure imperatorin suspension (Free IPT), both adjusted to an IPT concentration of 0.4 mg·mL^−1^, were loaded into preprocessed dialysis bags with a molecular weight cutoff of 14,000 Da.

These sealed dialysis bags were fully immersed in 40 mL of two separate dissolution media, namely SGF and SIF (containing 1.0% Tween 80) [60]. Then, the samples were placed in a thermostatic shaker maintained at 37 °C with constant shaking at 150 rpm. During the whole release process, 1 mL of liquid was collected from each dissolution medium at every preset time point, and we immediately supplemented each system with an identical volume of pre-warmed corresponding fresh medium to keep a constant liquid volume. All test samples were analyzed by injection under the chromatographic conditions established for the IPT standard curve, and the cumulative release–time curves for SGF and SIF systems were plotted separately.

### 3.6. Cellular Uptake and Transport Experiments

#### 3.6.1. Cell Culture

Caco-2 and HT29-MTX-E12 cells shared the same incubation setup: 37 °C within a humidified incubator filled with 5% CO_2_. We used DMEM as the basal medium, adding 10% fetal bovine serum, 1% non-essential amino acids, and 1% penicillin–streptomycin blend. When monolayers reached around 90% confluency, cells were subcultured, and then the obtained cell layers were utilized for subsequent cellular uptake and transmembrane transport tests.

#### 3.6.2. Preparation of C6@NLCs

Following the preparation method employed for the IPT@NLCs, 1 mg of C6 was dissolved in 40 mL of absolute ethanol, and the mixture was thoroughly vortexed to ensure complete dissolution of C6. Then, 10 mL of this C6-ethanol solution was added to the oil phase, while the stirring temperature of the oil phase was increased to 90 °C to remove ethanol. The other formulation parameters and processes remained unchanged, and thus C6@NLCs could be prepared.

#### 3.6.3. Cellular Uptake

Caco-2 and HT29-MTX-E12 cells at logarithmic proliferation stage were inoculated onto laser confocal culture dishes at a 7:3 cell proportion with fixed cell density, followed by overnight incubation [61]. Two parallel cell batches were prepared for subsequent testing: one undergoing mucus clearance while the other was kept intact without treatment. For mucus stripping, the original culture medium was fully aspirated, and cell monolayers were exposed to 10 mM of NAC solution for 30 min to clear mucus secreted by HT29-MTX-E12 cells [62]. After NAC processing, both mucus-depleted and untreated cell groups received three rounds of PBS rinsing separately. Then, the samples were cultured for 2 h in blank DMEM containing Free C6 or C6@NLCs (C6 concentration set to 0.8 μg·mL^−1^). Upon completion of nanoparticle incubation, cell surfaces were rinsed three times with PBS, fixed in 4% paraformaldehyde for 15 min, and then stained with 10 μg·mL^−1^ Hoechst 33,342 for 10 min, before a final thorough PBS wash. Processed culture dishes were transferred to a confocal laser scanning microscopy (CLSM; Leica, Wetzlar, Germany) fitted with a 630× oil immersion objective for fluorescence observation. Two sets of optical parameters were adopted for image capture: 405 nm excitation paired with 465 nm emission for nuclear staining signals, and 488 nm excitation matched with 568 nm emission for C6 fluorescent signals. Multi-channel fluorescence snapshots collected under distinct wavelength channels were overlaid for comprehensive analysis.

#### 3.6.4. Uptake Mechanisms

Caco-2 cells and HT29-MTX-E12 cells in the logarithmic growth phase were seeded into 6-well plates at a quantity ratio of 7:3 and a certain density per well, followed by cultivation for 24 h. Then, 100 μM of verapamil, 1 mM of protamine sulfate, 20 μM of β-carotene, 20 μM of quercetin, and 1 mM of sodium azide were added, respectively, for pre-treatment for 1 h, while Blank medium solution was added to the Control group. After the pre-treatment, the cells were co-incubated with DMEM blank medium solution containing C6@NLCs along with the aforementioned cell inhibitors for 2 h. The cells were then digested and collected, washed 3 times with pre-cooled PBS, resuspended in 0.5 mL PBS buffer, stored in an ice box away from light, and detected by flow cytometry (BD Biosciences, San Jose, CA, USA).

#### 3.6.5. Transport Experiments

We harvested Caco-2 and HT29-MTX-E12 cells at logarithmic growth stage and seeded them onto polycarbonate Transwell^®^ filter inserts (Model 724001, pore size 3.0 μm, effective membrane area 1.13 cm^2^, Wuxi NEST Biotechnology Co., Ltd., Wuxi, China) at a 7:3 cell proportion. We filled the apical donor compartment with 0.5 mL of complete culture medium and added 1.5 mL of identical medium to the basolateral receiver compartment. Medium renewal was performed every two days within the initial 7 days of cultivation, followed by daily medium replacement after the seventh day. Before starting transport detection, we discarded the original medium, rinsed cell monolayers three times with pre-warmed HBSS buffer, and kept the plates at 37 °C for 30 min to complete cell balance.

Two groups of transmembrane transport tests were carried out separately to detect AP-BL and BL-AP permeation behavior. For apical-to-basolateral detection, 500 μL of HBSS loaded with Free IPT or IPT@NLCs samples was supplemented into the donor side, and 1.5 mL blank HBSS buffer was filled in the receiver compartment. For reverse basolateral-to-apical transport measurement, we added 1.5 mL of drug-containing HBSS to the receiver chamber, while 500 μL of pure blank HBSS was placed in the donor insert. All Transwell culture plates were incubated at 37 °C, and we extracted liquid samples from both chambers after 4 h of incubation. Collected samples were centrifuged at 15,000 rpm for 15 min, and then UPLC was adopted to quantify the IPT concentration in supernatants. The apparent permeability coefficient (P_app_) was calculated via the following formula:

(3) $$P_{app}=\frac{V}{S\times C}\times \frac{dC}{dt}$$

In this equation, *V* denotes the volume of the receiving compartment, *S* stands for the surface area of the polycarbonate membrane, *C* refers to the initial concentration within the donor compartment, *dC* represents the concentration variation measured in the receiving phase, and *dt* corresponds to the incubation duration.

### 3.7. Oxidative Stress Experiments

#### 3.7.1. Optimal Treatment Concentration Screening of IPT@NLCs in 3T3-L1 Cells

3T3-L1 was cultivated in high-glucose DMEM complete medium (10% fetal bovine serum, 1% penicillin–streptomycin) at 37 °C with 5% CO_2_ humidified air. Cells were subcultured when the monolayers reached around 90% confluency. Logarithmic-phase 3T3-L1 cells were trypsinized, diluted with fresh medium to proper density, and then seeded into 96-well plates (100 μL/well). After 24 h of incubation, the culture medium was replaced with fresh medium containing a series of gradient concentrations of IPT@NLCs. Following continuous treatment for 24 h, 100 μL of diluted CCK-8 working solution was added to each well. The plates were incubated at 37 °C in darkness for 2 h, and the absorbance of each well was measured at 450 nm using a microplate reader. Finally, the optimal non-cytotoxic concentration of IPT@NLCs against 3T3-L1 cells was determined for subsequent experiments.

#### 3.7.2. Cell Viability Assay

Following the culture and seeding procedures for 3T3-L1 cells, the cells were incubated in a culture incubator for 24 h. The culture medium was then discarded and replaced with fresh medium containing the corresponding treatments: 1000 μM NAC, Blank NLCs prepared and diluted using the same procedures as IPT@NLCs, 15 μM Free IPT, or IPT@NLCs at an equivalent IPT concentration of 15 μM. Except for the Control group, cells were co-incubated with 300 μM H_2_O_2_ and the corresponding treatments for 24 h to establish the oxidative stress model [63]. The model control consisted of cells treated with 300 μM H_2_O_2_ alone, while the Control group received blank medium without H_2_O_2_ or drugs [64]. After 24 h of co-incubation, the medium was discarded, 100 μL of diluted CCK-8 reagent was added to each well, and the plates were incubated at 37 °C in the dark for 0.5 h. The OD value of each well was then measured at 450 nm using a microplate reader.

#### 3.7.3. Determination of Oxidative Stress Levels in 3T3-L1 Cells

In accordance with the method described in Section 3.7.2, after co-incubation of H_2_O_2_ with the treatment groups for 24 h, the medium was discarded and cell samples were collected. The activities of SOD, CAT, and GSH-Px, as well as the content of MDA, were measured according to the manufacturer’s instructions.

### 3.8. In Vivo Pharmacokinetic Study

Ten male Sprague–Dawley (SD) rats were randomized into two test groups with five animals per group. All rats underwent a 12 h overnight fasting period before dosing, while unlimited drinking water was provided throughout this period. Animals received oral gavage of Free IPT (0.5% CMC-Na suspension) or IPT@NLCs at an equivalent IPT dose of 100 mg·kg^−1^. At predetermined time intervals after administration, roughly 0.3 mL of blood was sampled from the retro-orbital venous plexus. Collected blood was transferred into heparin-coated centrifuge tubes and spun at 3500 rpm for 10 min to isolate plasma fractions. For plasma sample pretreatment, 100 μL of plasma was mixed with 20 μL of osthole internal standard solution (40 μg·mL^−1^), 50 μL of 0.2% Triton X-100 and 830 μL of methanol. The blended liquid was fully vortex-mixed, and then centrifuged at 13,000 rpm over 15 min. The resulting supernatant was dried completely with a vacuum centrifugal concentrator, and the dry residue was redissolved in a tiny volume of methanol for subsequent UPLC detection. 

Plasma imperatorin quantification relied on an Agilent 1290 UPLC instrument (Agilent Technologies, Santa Clara, CA, USA) [65,66]. The ultraviolet detector wavelength was fixed at 300 nm, and each sample injection volume was set to 10 μL. Separation was performed on a Boltimate-C_18_ column (2.1 × 100 mm, 2.7 μm) maintained at 30 °C with a constant flow speed of 0.3 mL·min^−1^. The mobile phase consisted of water (Phase A) and methanol (Phase B), operated under the gradient elution sequence listed below: 55% A (0–1.5 min); linear ramp to 70% A (1.5–5.5 min); linear ramp to 80% A (5.5–6.5 min); linear ramp to 88% A (6.5–6.6 min); and hold 88% A (6.6–10 min). Calibration curves were generated by taking the IPT concentration as the horizontal coordinate, and the peak area ratio between IPT and the osthole internal standard as the vertical coordinate. The linear fitting formula was A = 0.4471C − 0.0025, with a correlation coefficient of R = 0.9997, revealing favorable linearity within the concentration range of 43.50–17,400.00 μg·L^−1^.

### 3.9. Gastrointestinal Safety Assay

We divided male SD rats into two treatment groups, and 20 mg·kg^−1^ of Free IPT or IPT@NLCs was given via oral gavage every other day for four weeks; the Control group received equal sterile saline by the same oral route. H&E staining was applied to assess gastrointestinal irritation of the formulations in vivo. After 4 weeks of administration, we euthanized all rats. Stomach and small intestinal tissues were isolated, rinsed with saline, fixed in 4% paraformaldehyde, and paraffin-embedded. Sectioned tissues were subjected to H&E staining, and histological images were captured using an upright light microscope.

### 3.10. Intestinal Distribution Studies

We tracked the in vivo intestinal distribution of NLCs by orally giving rats Free C6 or C6@NLCs at an identical dose of 10 mg·kg^−1^. Two hours post-administration, we euthanized and dissected all rats to harvest intestinal tissue samples. Tissues were snap-frozen, sectioned into 5 μm slices, and mounted on glass slides. We counterstained these 5 μm frozen sections with DAPI for 5 min, and then captured fluorescence signals via confocal laser scanning microscopy.

### 3.11. Statistical Analysis

Every experiment was performed with three biological replicates, and all measurement data are expressed as mean ± standard deviation (SD). SPSS 17.0 was adopted to complete intergroup significance evaluation, while DAS 2.0 was utilized to calculate relevant pharmacokinetic indices. Intergroup comparisons relied on Student’s *t*-test, and discrepancies with *p* < 0.05 were judged to possess statistical significance.

## 4. Conclusions

In this study, a stable and high-performance oral delivery system of IPT@NLCs was successfully constructed using natural grape seed oil as the liquid lipid component. The optimized IPT@NLCs exhibited uniform particle size distribution, excellent colloidal stability, and high drug encapsulation efficiency, along with significantly enhanced in vitro drug release profiles compared to Free IPT. Owing to their unique lipid nanostructure and favorable mucus-penetrating properties, the NLCs were able to overcome both physical and biological barriers in the intestine, resulting in marked improvements in cellular internalization and transmembrane permeability for IPT. Mechanistic studies indicated that the enhanced intestinal absorption of IPT@NLCs was largely dependent on energy-mediated active transport and adsorptive endocytosis. Additionally, IPT@NLCs attenuated oxidative cell damage by elevating the activities of key antioxidant enzymes, thus exhibiting a synergistic antioxidative effect. Notably, in vivo pharmacokinetic data confirmed that the NLC formulation significantly boosted the oral bioavailability of IPT while causing no gastrointestinal irritation. Overall, these findings demonstrate that grape seed oil-based NLCs serve as a safe, green, and efficient oral delivery system for poorly soluble natural bioactives, offering robust evidence to support the future development and application of imperatorin in functional foods and oral drug products.

## Figures and Tables

**Table 1:** Physicochemical characterization of the Blank NLCs and IPT@NLCs (mean ± SD, n = 3).

Sample	Size (nm)	PDI	Zeta Potential (mV)	DL (%)	EE (%)
Blank NLCs	162.72 ± 2.38	0.174 ± 0.015	−21.40 ± 0.10	—	—
IPT@NLCs	186.63 ± 1.65	0.188 ± 0.008	−18.60 ± 0.30	9.08 ± 0.23	99.54 ± 0.10

**Table 2:** Kinetic model fitting for in vitro drug release profiles of IPT in SGF and SIF: comparison of model parameters and correlation coefficients (R^2^).

Sample		Zero Order	Higuchi	Korsmeyer–Peppas	
		R^2^	R^2^	R^2^	n
SGF	Free IPT	0.5558	0.9574	0.9095	0.4174
	IPT@NLCs	0.6500	0.9651	0.9945	0.8042
SIF	Free IPT	0.8078	0.9590	0.9766	0.3492
	IPT@NLCs	0.7234	0.9551	0.9959	0.9047

**Table 3:** The permeability and efflux ratio of Free IPT and IPT@NLCs (mean ± SD, n = 3).

Sample	P_app_ × 10^−5^ (cm·s^−1^)		Efflux Ratio P_app_(BL → AP)/P_app_(AP → BL)
	AP → BL	BL → AP	
Free IPT	1.269 ± 0.060	1.092 ± 0.071	0.861
IPT@NLCs	0.620 ± 0.019 **	1.607 ± 0.053 **	2.592

** *p* < 0.01 vs. Free IPT.

**Table 4:** Pharmacokinetic parameters after intragastric treatment with Free IPT and IPT@NLCs (100 mg·kg^−1^, mean ± SD, n = 5).

Sample	C_max_(μg·L^−1^)	T_max_(h)	T_1/2_(h)	AUC_(0-t)_(μg·L^−1^·h)	AUC_(0-∞)_(μg·L^−1^·h)	MRT(_0-t_)(h)	MRT_(0-∞)_(h)
Free IPT	942.37 ± 196.88	1.60 ± 0.22	4.55 ± 2.42	4396.21 ± 1136.77	4502.30 ± 1097.06	4.59 ± 0.60	5.33 ± 1.06
IPT@NLCs	5101.45 ± 321.48 **	1.90 ± 0.22	4.07 ± 0.52	41,091.61 ± 7649.75 **	41,972.51 ± 8073.20 **	6.05 ± 0.65 **	6.38 ± 0.72

** *p* < 0.01 vs. Free IPT.

## Data Availability

The original contributions presented in this study are included in the article. Further inquiries can be directed to the corresponding authors.
